# Fluorescent TEM-1 β-lactamase with wild-type activity as a rapid drug sensor for *in vitro* drug screening

**DOI:** 10.1042/BSR20140057

**Published:** 2014-09-05

**Authors:** Wing-Lam Cheong, Ming-San Tsang, Pui-Kin So, Wai-Hong Chung, Yun-Chung Leung, Pak-Ho Chan

**Affiliations:** *State Key Laboratory of Chirosciences, Food Safety and Technology Research Centre, Department of Applied Biology and Chemical Technology, The Hong Kong Polytechnic University, Hung Hom, Kowloon, Hong Kong, People's Republic of China; †The Hong Kong Polytechnic University Shenzhen Research Institute, Shenzhen 518057, People's Republic of China

**Keywords:** antibiotics, bacteria, β-lactamase, drug screening, inhibitors, sensor, BAEE, *N^c^*-benozyl-L-arginine ethyl ester, ES, enzyme-substrate, ES*, covalent enzyme-substrate, ESI–MS, electrospray ionization mass spectrometry, ESBL, extended spectrum β-lactamase, IRT, inhibitor-resistant TEM, TIPP, tetraiodophenolphthalein, TEM, transmission electron microscopy

## Abstract

We report the development of a novel fluorescent drug sensor from the bacterial drug target TEM-1 β-lactamase through the combined strategy of Val^216^→Cys^216^ mutation and fluorophore labelling for *in vitro* drug screening. The Val^216^ residue in TEM-1 is replaced with a cysteine residue, and the environment-sensitive fluorophore fluorescein-5-maleimide is specifically attached to the Cys^216^ residue in the V216C mutant for sensing drug binding at the active site. The labelled V216C mutant has wild-type catalytic activity and gives stronger fluorescence when β-lactam antibiotics bind to the active site. The labelled V216C mutant can differentiate between potent and impotent β-lactam antibiotics and can distinguish active-site binders from non-binders (including aggregates formed by small molecules in aqueous solution) by giving characteristic time-course fluorescence profiles. Mass spectrometric, molecular modelling and trypsin digestion results indicate that drug binding at the active site is likely to cause the fluorescein label to stay away from the active site and experience weaker fluorescence quenching by the residues around the active site, thus making the labelled V216C mutant to give stronger fluorescence in the drug-bound state. Given the ancestor's role of TEM-1 in the TEM family, the fluorescent TEM-1 drug sensor represents a good model to demonstrate the general combined strategy of Val^216^→Cys^216^ mutation and fluorophore labelling for fabricating tailor-made fluorescent drug sensors from other clinically significant TEM-type β-lactamase variants for *in vitro* drug screening.

## INTRODUCTION

β-Lactam antibiotics have been widely used as therapeutic agents in the treatment of bacterial infections. These drugs, which share a common structural characteristic of four-membered β-lactam ring, can irreversibly bind to the active site of penicillin-binding proteins, thus inhibiting these bacterial proteins from synthesizing cell walls and leading to cell death [1]. The overuse of β-lactam antibiotics in various areas (e.g. clinical treatment and animal farming), however, has led to a worrying worldwide clinical problem–the emergence of antibiotic-resistant bacteria. Many bacteria have developed an effective defence mechanism against β-lactam antibiotics by producing β-lactamases, which are efficient enzymes capable of catalysing β-lactam hydrolysis [2,3]. The TEM family is classified as class A serine-type β-lactamase in which the Ser^70^ residue in the active site is activated by Glu^166^ to form Ser^70-O^−^^ (through the deprotonation of the side-chain–OH group), which subsequently opens the β-lactam ring through the acylation with the β-lactam carbonyl group [4]. TEM-1 is regarded as the ancestor of the TEM family and offers ampicillin resistance to pathogenic bacteria (e.g. 90% of ampicillin resistance to *Escherichia coli* [5]; ampicillin and penicillin resistance to *Haemophilus influenzae* and *Neisseria gonorrhoeae* [6]). To date, more than 100 TEM variants have been derived from TEM-1 through one or more amino acid mutations, including clinically relevant ESBLs (extended spectrum β-lactamases) and IRT (inhibitor-resistant TEM) β-lactamases (http://www.lahey.org/Studies/). The emergence of such enzymes has compromised the clinical utility of a broad spectrum of β-lactam antibiotics, including penicillins, cephalosporins and β-lactamase inhibitors [2,7–11]. At present, TEM variants (e.g. TEM-3, TEM-10, TEM-26 and TEM-52) are still widespread in many countries [9,10]. In view of this clinical threat, development of new and potent β-lactam antibiotics and non-β-lactam inhibitors against TEM-type β-lactamases has been a very important research topic [12]. In recent years, the advent of computational drug screening has facilitated the discovery of new drug candidates through the high-throughput screening of compounds in chemical libraries *in silico* [13–16]. Despite this, *in vitro* drug screening is still an indispensable task because this study gives valuable experimental information on protein–drug binding in solution and the efficacy of drug candidates [17,18]. Nitrocefin, a colorimetric β-lactam antibiotic, has been routinely used to assess the inhibitory function of new drugs against β-lactamases [19]. This colorimetric antibiotic acts as a competitive binder to β-lactamases to probe the inhibitory activity of drug candidates; the enzymatic hydrolysis and subsequent coloured product formation (with strong absorbance at 482 nm) of nitrocefin will be reduced if the drug candidates can bind to the active site of β-lactamases, and vice versa. The nitrocefin method, however, is an indirect approach as it is unable to directly probe the binding interaction of drug candidates with the active site of β-lactamases. Probing β-lactamase-drug binding, in fact, provides valuable information for new drug development (e.g. the binding affinity of new β-lactam antibiotics/inhibitors and their inhibitory activities). Despite the clinical relevance of many TEM-type β-lactamases, no attempt has been made to develop drug sensors based on such important molecular drug targets. We reasoned that TEM-type β-lactamases can be converted into fluorescent drug sensors through site-specific cysteine incorporation and fluorescent modification for *in vitro* drug screening purposes. Unlike the construction of fluorescently labelled proteins as simple ligand-binding biosensors [20–26], the development of fluorescent drug sensors from TEM-type β-lactamases for *in vitro* drug screening is much more challenging because the catalytic activity of the modified β-lactamases must be largely conserved in order to mimic their wild-type form for drug testing purposes. In this regard, it is very critical to choose a suitable residue in the target protein structure (for cysteine replacement) that does not significantly interfere with the enzymatic activity but still allows the attached fluorescent probe to sense drug binding at the active site.

**Figure 1:**
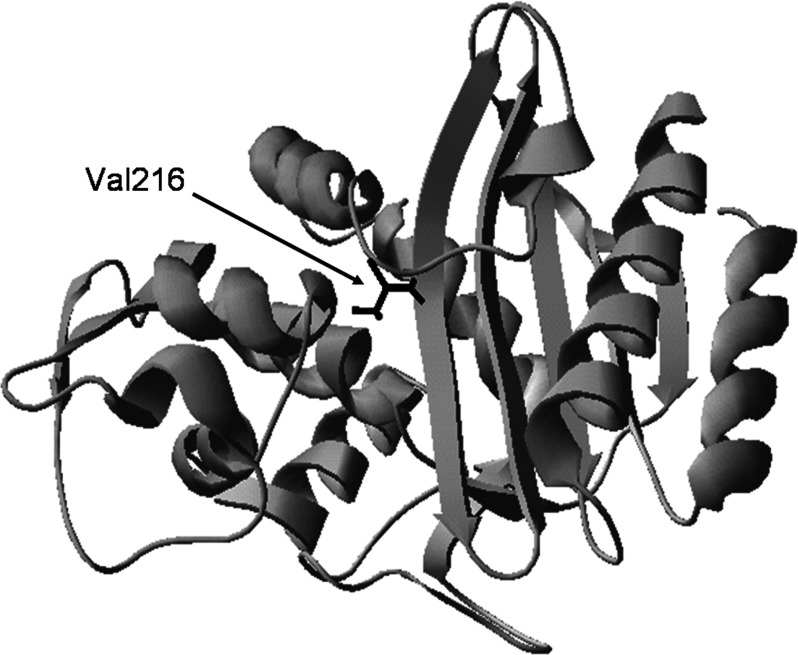
Structure of the TEM-1 β-lactamase The Val^216^ residue is shown in black. The protein structure was constructed from TEM-1 (PDB code: 1ZG4).

Herein, we describe the development of a rapid fluorescent drug sensor from the clinically relevant TEM-1 β-lactamase for *in vitro* drug screening. We chose TEM-1 because this enzyme is regarded as the ancestor in the TEM family in which many ESBL and IRT variants have been derived through one or more amino acid mutations in TEM-1 [2,6,8,9]. Thus, TEM-1 represents a good protein model to demonstrate the general strategy for developing fluorescent drug sensors from other clinically relevant TEM-type β-lactamases. Our strategy is to replace the Val^216^ residue in TEM-1 with a cysteine to produce the V216C mutant and then covalently attach an environment-sensitive fluorescent probe (fluorescein-5-malemide) to Cys^216^ (through the formation of a thioether bond) for detecting drug binding at the active site [20,21]. The Val^216^ residue was chosen because it is a non-catalytic residue which lies at the upper part of the active site (Figure 1). Thus, the replacement of Val^216^ with a cysteine and the subsequent fluorophore labelling can largely conserve the catalytic activity of TEM-1, while the attached fluorophore can sensitively detect drug binding at the active site. This fluorescent TEM-1 V216C mutant with wild-type activity can mimic the catalytic behaviour of wild-type TEM-1, thus allowing itself to serve as an *in vitro* drug screening tool. More importantly, Val^216^ is highly conserved in TEM-type β-lactamases. Thus, the combined strategy of Val^216^→Cys^216^ mutation and fluorophore labelling represents a general approach to fabricating tailor-made fluorescent drug sensors from other clinically relevant TEM variants for *in vitro* drug-screening purposes.

## EXPERIMENTAL

### Chemicals and instruments

Penicillin G, ampicillin, cefoxitin (sodium salt), potassium clavulanate, BAEE (*N^c^*-benozyl-L-arginine ethyl ester) hydrochloride, Congo red, 3′,3″,5′,5″-TIPP (tetraiodophenolphthalein), trypsin and aspirin were purchased from Sigma–Aldrich. Fluorescein-5-maleimide was purchased from Invitrogen. Fluorescence experiments were performed on a PerkinElmer LS-50B spectrofluorimeter. PerkinElmer Lambda 35 UV/VIS spectrophotometer was used for enzyme kinetic studies. ESI–MS (electrospray ionization mass spectrometry) experiments were conducted using a micromass Q-Tof-2™ spectrometer. CD measurements were performed on a JASCO J-810 spectropolarimeter. TEM studies were performed using a DEOL JEM-2010 electron microscope.

### Protein expression and purification

*E. coil* BL21(DE3) was used as the bacterial strain to produce the wild-type and mutant (V216C) forms of the TEM-1 β-lactamase. The proteins were (His)_6_-tagged at their C-terminus. The bacterial strain was incubated in 2×TY medium overnight at 37°C with shaking at 280 rev/min. A 2-ml portion of the overnight culture was then inoculated into 200 ml of sterile 2×TY medium. The bacterial culture was further incubated at 37°C with shaking at 280 rev/min. When OD_600_ reached 0.8–0.9, IPTG (isopropyl β-D-thiogalactoside) was then added to the bacterial culture to induce protein expression. The bacterial culture was then incubated at 37°C with shaking at 280 rev/min for another 6 h. The cell pellet was collected by centrifugation and then lysed by sonication. The proteins in the supernatant were purified by Ni^2+^-affinity chromatography with a linear gradient elution of 0–0.5 M imidazole in 20 mM sodium phosphate buffer (pH 7.0). The purified proteins were dialysed against deionized H_2_O at 4°C, freeze-dried and stored at −20°C. The purity of the proteins was examined by SDS/PAGE.

### Fluorophore labelling

The V216C mutant was labelled with fluorescein-5-maleimide. The protein was first dissolved in 6 ml of 50 mM potassium phosphate buffer (pH 7.0). A 5-fold molar excess of fluorescein-5-maleimide [20 mM, dissolved in DMF (dimethylformamide)] was then added to the protein solution, and the protein solution was stirred in dark at 25°C for 30 min. After labelling, the protein solution was dialysed against deionized H_2_O at 4°C and then freeze-dried.

### CD measurements

The far-UV CD spectra of the wild-type TEM-1 enzyme and the labelled and unlabelled V216C mutants were recorded using a JASCO J-810 spectropolarimeter. The wild-type TEM-1 enzyme and the labelled and unlabelled V216C mutants were dissolved in 50 mM potassium phosphate buffer (pH 7.0) to 5 μM. The instrument was purged with nitrogen for 30 min before CD measurements. A quartz cuvette of 0.1-cm light absorption path length was used for far-UV (190–250 nm) CD measurements. The CD spectra were recorded in triplicate for each protein.

### Enzyme kinetics studies

The catalytic activities of the wild-type TEM-1 enzyme and the labelled and unlabelled V216C mutants were studied by the spectrophotometric method. The enzyme kinetics assays were performed with 10–1000 μM penicillin G and ampicillin as the substrates in 50 mM potassium phosphate buffer (pH 7.0) at 20°C. The hydrolytic reaction was initiated by mixing the enzymes with the substrates in a quartz cuvette of 1-cm light absorption path length. The UV absorbance of penicillin G and ampicillin were then monitored at 233 and 235 nm, respectively, at each substrate concentration as a function of time. The initial rate for each substrate concentration was determined in duplicate by analysing the absorbance over the first 60 s. The initial rates were then fitted to the Hanes–Woolf equation to determine the *k*_cat_ and *K_m_* values.

### Fluorescence measurements

The fluorescence of the labelled V216C mutant (20 nM) in the absence and presence of drug compounds (0, 0.01, 0.1 and 1 mM cefoxitin, penicillin G, ampicillin and clavulanate) in 50 mM potassium phosphate buffer (pH 7.0) was monitored using a PerkinElmer LS-50B spectrofluorimeter. A quartz cuvette of 1-cm light absorption path length was used for fluorescence measurements. The excitation and emission slit widths were 5 nm. The fluorescence signal of the labelled V216C mutant at 515 nm was recorded with excitation at 494 nm.

### MS studies

#### (a) Molecular mass measurements

The molecular masses of the wild-type TEM-1 enzyme and the labelled and unlabelled V216C mutants were determined by ESI–MS. Each of the proteins was dissolved in 150 μl of 20 mM ammonium acetate buffer (pH 7.0) to a final concentration of 5–10 μM. The protein solutions were centrifuged to remove insoluble materials. ESI–MS measurements were performed on a Micromass Q-Tof-2™ spectrometer. The protein samples were mixed with 1% formic acid dissolved in acetonitrile (1: 1, v/v) and injected into the electrospray source by a syringe pump (Harvard Apparatus, model 22) at a flow rate of 5 μl min^−1^. The mass spectrometer was scanned over a range of 700–1600 *m*/*z* and the multiply charged protein ion peaks were detected. The capillary and cone voltage were set at 3 kV and 30 V, respectively. Nitrogen was used as the desolvation, cone and nebulizing gas. The nebulizing gas was fully opened. The flow rates of the desolvation gas and cone gas were set at 400 and 50 L·h^−1^, respectively. The *m*/*z*-axis was calibrated externally with 10 μM horse heart myoglobin (*M*_α_=16950.5 Da). The raw multiply charged spectra were deconvoluted by the MassLynx 4.1 Transform Program.

#### (b) Detection of ES (enzyme–substrate) complexes

The formation of ES* (covalent enzyme–substrate) complex during the course of hydrolytic reaction was probed by ESI–MS. A 200-μl portion of the labelled V216C mutant (50 μM) was added to 200 μl of cefoxitin (100 mM), and the mixture was made up to 2000 μl with 20 mM ammonium acetate solution (pH 7.0). At different time intervals, a 400-μl portion of the reaction mixture was pipetted to 400 μl of 20 mM ammonium acetate with 2% (v/v) formic acid to quench the hydrolytic reaction (pH~2). The mixture was then concentrated and buffer-exchanged [20 mM ammonium acetate with 2% (v/v) formic acid] 1–2 times by means of Amicron® Ultra-15 (NMWL=10 000) centrifugal filter devices (Millipore) to a final volume of about 150 μl. ESI–MS measurements were then performed on a Micromass Q-Tof-2™ spectrometer. Concentrated protein samples were mixed with 1% formic acid dissolved in acetonitrile (1: 1 v/v) and injected into the electrospray source by a syringe pump (Harvard Apparatus, model 22) at a flow rate of 5 μl·min^−1^. The mass spectrometer was scanned over a range of 700–1600 *m*/*z*, and the multiply charged protein ion peaks were detected. The capillary and cone voltage were set at 3 kV and 30 V, respectively. Nitrogen was used as the desolvation, cone and nebulizing gas. The nebulizing gas was fully opened. The flow rates of the desolvation gas and cone gas were set at 400 and 50 L·h^−1^, respectively. The *m*/*z*-axis was calibrated externally with 10 μM horse heart myoglobin (*M*_α_=16950.5 Da). The raw multiply charged spectra were deconvoluted by the MassLynx 4.1 Transform Program.

### Molecular modelling studies

Molecular modelling of the labelled V216C mutant with and without penicillin G was performed using ICM-Pro 3.4-8a (Molsoft). The molecular model of the V216C mutant was constructed from the structure of TEM-1 (PDB: 1FQG). BPMC (Biased Probability Monte Carlo) minimization was used for energy minimization.

### TEM (transmission electron microscopy) measurements

TEM was used to examine the formation of ‘drug aggregates’ by small molecules under aqueous conditions. Samples of 10 μM Congo red (dissolved in deionized water) and TIPP (dissolved in 0.5% DMSO) were incubated with the labelled V216C mutant (20 nM). A drop of the solution mixture (10 μl) was then applied onto a carbon grid (400 mesh), stained with 0.5% PTA (phosphotungstic acid) and then dried overnight. TEM pictures were obtained with a 200 kV DEOL JEM-2010 electron microscope equipped with a Gatan MSC 794 CCD camera.

### Trypsin digestion studies

The labelled V216C mutant (0.2 mg·ml^−1^) was mixed with trypsin (0.01 mg·ml^−1^) in 50 mM potassium phosphate buffer (pH 7.0) in a quartz cuvette of 1-cm light absorption path length. The fluorescence spectra of the labelled V216C mutant were then recorded at various time intervals (*t*=0, 2, 4, 6 and 8 h) using a PerkinElmer LS50B spectrofluorimeter. The excitation wavelength was 460 nm, and the excitation and emission slit widths were 2.5 nm.

For SDS/PAGE analysis, the labelled V216C mutant (2 mg·ml^−1^) was mixed with trypsin (0.1 mg·ml^−1^) in 50 mM potassium phosphate buffer (pH 7.0) for 8 h. After digestion, a 10-μl portion of the reaction mixture was collected and loaded onto a SDS/PAGE gel. The SDS/PAGE gel was illuminated with the UV light to obtain a fluorescent image. For comparison, a 10-μl portion of the labelled V216C mutant (2 mg·ml^−1^) without trypsin digestion was also analysed in the same SDS/PAGE assay.

## RESULTS AND DISCUSSION

### Characterization of the labelled TEM-1 V216C mutant

**Figure 2:**
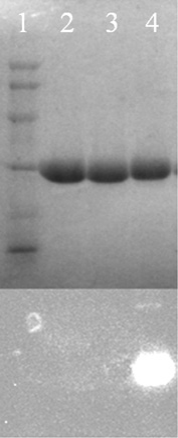
SDS/PAGE analysis of the wild type and mutant forms of the TEM-1 β-lactamase Lane 1 (protein markers): rabbit muscle phosphorylase b (97400 Da), BSA (66200 Da), hen egg white ovalbumin (45 000 Da), bovine carbonic anhydrolase (31 000 Da), SBTI (soy-bean trypsin inhibitor, 21 500 Da), hen egg white lysozyme (14 400 Da); Lane 2: wild-type TEM-1; Lane 3: unlabelled V216C incubated with fluorescein (without the maleimide linker); Lane 4: labelled V216C. The fluorescent image (bottom) was obtained by illuminating the SDS/PAGE gel (top) with the UV light.

The labelling reaction of the TEM-1 V216C mutant with the thiol-reactive fluorophore fluroescein-5-maleimide was monitored by SDS/PAGE. Briefly, wild-type TEM-1 and the V216C mutant were incubated with fluorescein-5-maleimide and then purified by dialysis to remove excess fluorophore. The protein samples were then loaded onto a SDS/PAGE gel for analysis. Figure 2 shows the protein bands for wild-type TEM-1 as well as the labelled and unlabelled V216C mutants. For the wild-type TEM-1 enzyme (without free cysteine), no observable fluorescent band appears upon illumination with the UV light (lane 2, Figure 2). In contrast, the labelled V216C mutant shows a strong fluorescent band (lane 4, Figure 2). These observations indicate that fluorescein-5-maleimide is specifically attached to the Cys^216^ residue in the V216C mutant. In order to verify that the fluorescein molecule is covalently bound to the Cys^216^ residue in the V216C mutant through the maleimide linker, a control experiment was performed in which the V216C mutant was incubated with fluorescein (without the maleimide linker) and then analysed by SDS/PAGE. In this case, the V216C mutant remains non-fluorescent (lane 3, Figure 2). This observation reveals that the fluorescein molecule is covalently bound to the Cys^216^ residue through the thiol-reactive maleimide linker.

The labelled V216C mutant was further analysed by UV–Vis spectrophotometry. Supplementary Figure S1 (available at http://www.bioscirep.org/bsr/034/bsr034e136add.htm) shows the UV–Vis spectrum of the labelled V216C mutant. The labelled V216C mutant exhibits a visible absorption peak at 494 nm, which corresponds to the light absorption by fluorescein. This observation is consistent with the fact that the V216C mutant is covalently linked to fluorescein-5-maleimide after fluorophore labelling (Figure 2). To examine the extent of fluorophore labelling on the V216C mutant, we analysed the mass values of the V216C mutant before and after labelling with fluorescein-5-maleimide using ESI–MS. Supplementary Figure S2 (available at http://www.bioscirep.org/bsr/034/bsr034e136add.htm) shows the ESI mass spectra of the unlabelled and labelled V216C mutants. The unlabelled V216C mutant shows a mass peak of 29898 Da (Figure S2A). After labelling with fluorescein-5-maleimide, the V216C mutant gives a new mass peak of 30324 Da (Figure S2B). The mass difference between the labelled and unlabelled V216C mutants is 426 Da, which is close to the molecular mass of fluorescein-5-maleimide (427 Da). Furthermore, no significant mass peak appears at 29898 Da (corresponding to the unlabelled V216C mutant) in the mass spectrum of the labelled V216C mutant, indicating that the V216C mutant is completely labelled with fluorescein-5-maleimide (Figure S2B).

Far-UV CD measurements were performed on wild-type TEM-1 as well as the unlabelled and labelled V216C mutants. Supplementary Figure S3 (available at http://www.bioscirep.org/bsr/034/bsr034e136add.htm) shows the resulting far-UV CD spectra. In all cases, the CD spectra show strong peaks at 210 and 220 nm, which are characteristic of α-helical structure (Figure S3). The CD signal for the labelled V216C mutant is very similar to those of the unlabelled V216C mutant and wild-type TEM-1, indicating that the secondary structure of the labelled V216C mutant is virtually similar to those of the unlabelled mutant and wild-type TEM-1.

### Enzyme kinetics studies

The catalytic activities of wild-type TEM-1 as well as the unlabelled and labelled V216C mutants were investigated by the spectrophotometric method [27–29]. With ampicillin as the substrate, the *k*_cat_ value for the unlabelled V216C mutant is similar to that of wild-type TEM-1, indicating that the V216C mutant has similar catalytic activity to wild-type TEM-1 after the V216C mutation (Table 1). Interestingly, the labelled V216C mutant also has similar catalytic activity compared to the wild-type TEM-1 enzyme (similar *k*_cat_) even though the mutant carries the fluorescein molecule at its active site (Table 1). Similar observations were also obtained with penicillin G as the substrate. In all cases, the *k*_cat_ values for the unlabelled and labelled V216C mutants and the wild-type TEM-1 enzyme are within the same order of magnitude (Table 1). Unlike the case of removing catalytic residues (e.g. Glu^166^) in class A β-lactamases, which results in more than 1000-fold decrease in *k*_cat_ [21,27,30], the replacement of Val^216^ with a cysteine in the TEM-1 structure does not significantly impair the catalytic activity of the enzyme. This phenomenon can be attributed to the fact that the Val^216^ residue at the upper part of the active site is not directly involved in the catalytic process. Thus, the V216C mutation and the subsequent fluorophore labelling allow the catalytic activity of the labelled V216C mutant to be largely conserved with respect to that of the wild-type TEM-1 enzyme. With its conserved catalytic activity, the labelled V216 mutant can mimic the natural drug target, the wild-type TEM-1 enzyme, for *in vitro* drug screening purposes.

**Table 1:** Steady-state kinetic parameters of the wild-type and mutant forms of the TEM-1 β-lactamase

Form	*k*_cat_ (s^−1^)	*K_M_* (M)	*k*_cat_/*K_M_* (s^−1^·M^−1^)
Ampicillin			
Wild-type	1040±34	(6.5±1.9)×10^−5^	(1.6±0.5)×10^7^
V216C	873±13	(1.4±0.1)×10^−4^	(6.3±0.1)×10^6^
Labelled V216C	650±58	(7.0±0.8)×10^−4^	(9.2±1.9)×10^5^
Penicillin G			
Wild-type	746±31	(1.3±0.2)×10^−4^	(5.5±1.2)×10^6^
V216C	491±13	(1.6±0.2)×10^−4^	(3.0±0.4)×10^6^
Labelled V216C	148±9	(2.6±0.4)×10^−4^	(5.6±1.2)×10^5^

### Fluorescence response of the labelled V216C mutant to drug binding

**Figure 3:**
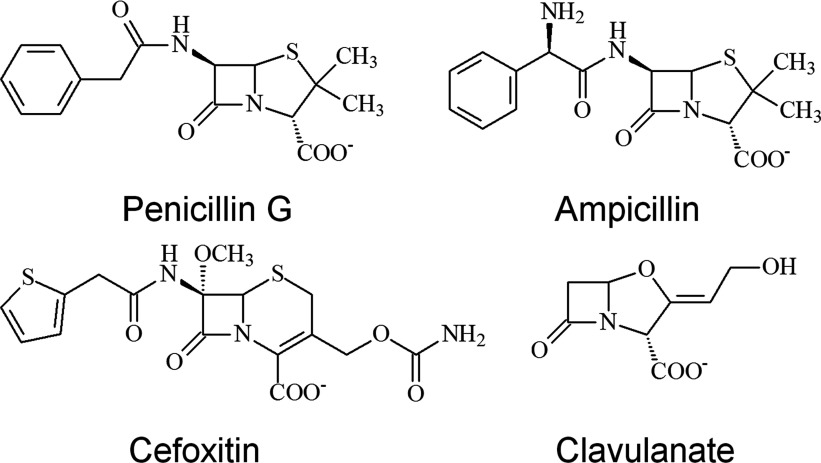
Chemical structures of the β-lactam antibiotics and β-lactamase inhibitor used in this study

**Figure 4:**
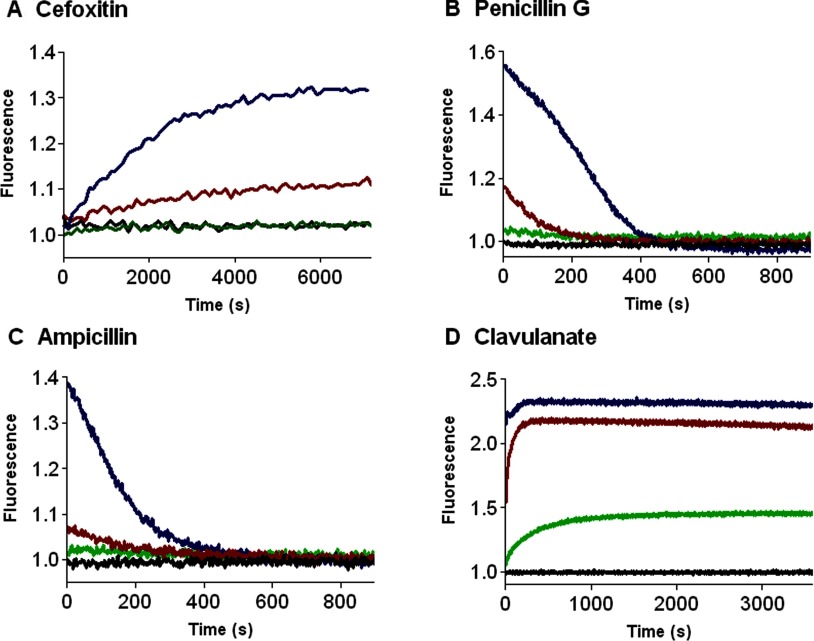
Time-course fluorescence measurements of the labelled V216C mutant with different concentrations of β-lactam antibiotics and β-lactamase inhibitor (**A**) cefoxitin; (**B**) penicillin G; (**C**) ampicillin; (**D**) clavulanate. The concentrations of the β-lactam antibiotics and β-lactamase inhibitor are shown in different colours: 0 mM (black), 0.01 mM (green), 0.1 mM (red), and 1 mM (blue). The labelled V216C mutant (20 nM) was dissolved in 50 mM potassium phosphate buffer (pH 7.0).

The ability of the labelled V216C mutant to perform *in vitro* drug screening was investigated by fluorescence spectroscopy. The β-lactam antibiotics and β-lactamase inhibitor (clavulanate) used in this study are shown in Figure 3. We examined the fluorescence response of the labelled V216C mutant with cefoxitin, which is a cephalosporin antibiotic with strong resistance to the hydrolytic activity of TEM-1 [31–33]. Figure 4(A) shows the fluorescence signals of the labelled V216C mutant at different time intervals with different concentrations of cefoxitin. In the absence of cefoxitin, the labelled V216C mutant does not show significant fluorescence changes over the time course (Figure 4A). Upon addition of cefoxitin, the fluorescence of the labelled V216C mutant increases as a function of time and then becomes sustained (Figure 4A). Moreover, the fluorescence enhancement of the labelled V216C mutant increases with the concentration of cefoxitin (Figure 4A).

The fluorescence response of the labelled V216C mutant with less-resistant β-lactam antibiotics was also studied. Figure 4(B) shows the fluorescence signals of the labelled V216C mutant with penicillin G at different time intervals. Without penicillin G, the labelled V216C mutant just gives steady and weak fluorescence over the time course (Figure 4B). In the presence of penicillin G (1 mM), the fluorescence of the labelled V216C mutant increases instantaneously and then declines (Figure 4B). With a lower concentration of penicillin G (0.1 mM), the fluorescence signal of the labelled V216C mutant also increases instantaneously (but to a smaller extent) and then decreases gradually (Figure 4B). Similar fluorescence measurements were also performed on the labelled V216C mutant with ampicillin. As similar to the case of penicillin G, the fluorescence of the labelled V216C mutant increases rapidly at the initial stage and then declines gradually over the time course (Figure 4C). The fluorescence response of the labelled V216C mutant towards clavulanate was also investigated. Clavulanate is an effective inhibitor against many class A β-lactamases [31,32]. This inhibitor can irreversibly bind to the active site of class A β-lactamases to form a stable covalent complex, resulting in the inhibition of enzymatic hydrolysis [31,34,35]. The fluorescence signals of the labelled V216C mutant in the presence of different concentrations of clavulanate are shown in Figure 4(D). In the absence of clavulanate, the labelled V216C mutant does not show significant fluorescence changes over the time course (Figure 4D). Upon addition of clavulanate (0.01 mM), the fluorescence of the labelled V216C mutant increases as a function of time and then becomes sustained (Figure 4D). The labelled V216C mutant gives stronger fluorescence in the presence of higher concentrations of clavulanate (0.1 and 1 mM) (Figure 4D).

The specificity of the labelled V216C mutant was then examined. To this end, the fluorescence response of the labelled V216C mutant with non-binders (e.g. aspirin and BAEE) was monitored. As shown in Supplementary Figure S4 (available at http://www.bioscirep.org/bsr/034/bsr034e136add.htm), the fluorescence signals of the labelled V216C mutant with these non-binders remain virtually unchanged over the time course, unlike the cases of drug binders (e.g. cefoxitin, penicillin G, ampicillin and clavulanate; Figures 4A–D). These observations highlight the ability of the labelled V216C mutant to specifically recognize drug candidates capable of binding to the active site. The ability of the labelled V216C mutant to distinguish drug binders from ‘drug aggregates’ was also studied. Recent studies have shown that drug compounds selected *in silico* may form aggregates in aqueous solution [17,36–39]. Such ‘drug aggregates’ can inhibit β-lactamase activity *in vitro* through non-specific mechanisms (e.g. protein adsorption/absorption), thus leading to hitting false-positive drug compounds in drug screening [17,36–39]. To investigate whether the labelled V216C mutant can distinguish drug binders from ‘drug aggregates’, we studied the fluorescence response of the labelled V216C mutant in the presence of the aggregates formed by Congo red and TIPP. In aqueous solution, Congo red and TIPP tend to form aggregates, as revealed by the TEM images (Supplementary Figure S5 available at http://www.bioscirep.org/bsr/034/bsr034e136add.htm). Unlike the cases of drug binders (cefoxitin, penicillin G, ampicillin and clavulanate; Figures 4A–4D), the labelled V216C mutant does not show significant fluorescence changes with the aggregates formed by Congo red (Supplementary Figure S6A available at http://www.bioscirep.org/bsr/034/bsr034e136add.htm) and TIPP (Figure S6B). These observations indicate that the labelled V216C mutant can distinguish active-site-binding drugs from ‘drug aggregates’ *in vitro*.

Taking all the fluorescence results together, the labelled V216C mutant can specifically detect drug binders capable of binding to the active site and eliminate non-binders and ‘drug aggregates’ in *in vitro* drug screening. Furthermore, the labelled V216C mutant can differentiate β-lactamase-resistant antibiotics/inhibitors (e.g. cefoxitin and clavulanate) from β-lactamase-unstable antibiotics (e.g. penicillin G and ampicillin) by giving characteristic fluorescence profiles (Figure 4). These interesting observations highlight the potential use of the labelled V216C mutant as a rapid drug sensor for *in vitro* drug screening in the development of new β-lactam antibiotics/inhibitors.

### Turn-on effect of drug binding on the labelled V216C mutant

To investigate the origin of the fluorescence changes of the labelled V216C mutant, the catalytic reaction of the labelled V216C mutant with cefoxitin was analysed by ESI–MS. Class A β-lactamases (including TEM enzymes) catalyse the hydrolysis of β-lactam antibiotics according to the three-step model (below) (where E is the free enzyme, S is a substrate, ES is a non-covalent enzyme-substrate complex, ES* is a covalent enzyme-substrate complex, and P is the product) [40]:

$$E+S↔\mathrm{ES}\to {\mathrm{ES}}^{*}\to E+P$$

**Figure 5:**
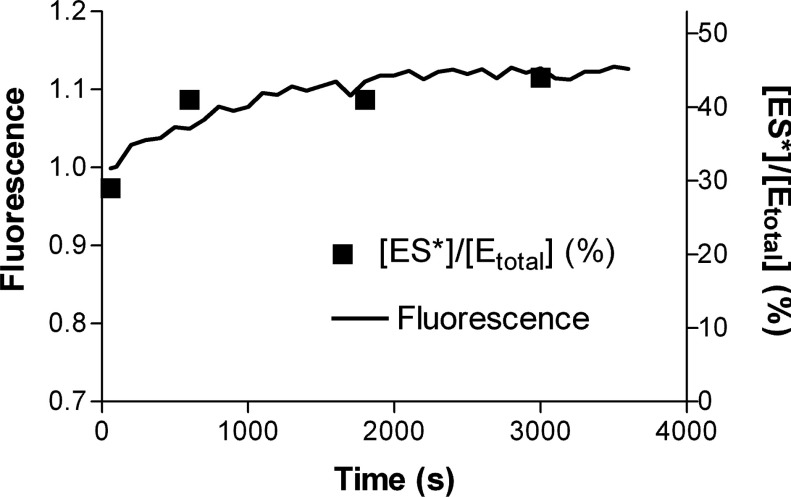
Time-course ESI–MS and fluorescence measurements on the binding of the labelled V216C mutant to cefoxitin The time-course fluorescence profile of the labelled V216C mutant and the [ES*]/[E_total_] values measured by ESI–MS at different time intervals are shown in a solid line and black squares, respectively. The reaction of the labelled V216C mutant with cefoxitin took place in 20 mM ammonium acetate solution (pH 7.0). [Labelled V216C]=5 μM; [Cefoxitin]=10 mM.

Thus, by addition of an acid to denature TEM-1 at different time intervals, the catalytic reaction will be quenched and therefore the free enzyme (E) and the ES* complex can be probed over the time course. In this MS study, we chose cefoxitin as the substrate because it is resistant to the hydrolytic action of TEM-type β-lactamases and can therefore form a stable ES* complex in which the hydroxyl group of Ser^70^ in the active site is covalently linked to the β-lactam carbonyl carbon with the aid of the activating residue Glu^166^ [31,33]. The resulting ES* complex is long-lived and can therefore be easily probed by ESI–MS. Supplementary Figure S7 (available at http://www.bioscirep.org/bsr/034/bsr034e136add.htm) shows the ESI mass spectra of the labelled V216C mutant with cefoxitin at different time intervals. The mass peaks A and B correspond to the free labelled V216C mutant (E) and the ES* complex, respectively. The relative population of ES* ([ES*]/[E_total_], where [E_total_]=[E]+[ES*]) at each time interval was then determined. The relative ES* population increases as a function of time, indicating that the ES* complex accumulates over the time course (rectangle, Figure 5). The relationship of the ES* formation with the fluorescence change of the labelled V216C mutant was further investigated. To this end, the fluorescence of the labelled V216C mutant with cefoxitin was monitored at different time intervals under the solution conditions for the ESI–MS measurements ([labelled V216C]=5 μM; [cefoxitin]=10 mM; 20 mM ammonium acetate, pH 7.0). Under such conditions, the fluorescence of the labelled V216C mutant increases as a function of time (Figure 5). Interestingly, the increasing fluorescence profile of the labelled V216C mutant is consistent with the increasing mass spectrometric profile of ES*, indicating that the fluorescence enhancement of the labelled V216C mutant arises from the formation of ES* complex (Figure 5).

We tried to probe the ES* complexes of the labelled V216C mutant with penicillin G and ampicillin by ESI–MS. However, the reaction was so fast under the solution conditions for ESI–MS measurements that the ES* complexes could not be traced. In order to unravel the origin of the initial fluorescence enhancements and the subsequent fluorescence declines of the labelled V216C mutant with penicillin G and ampicillin (Figures 4B–4C), we performed time-course UV absorbance measurements on these antibiotics and then compared these results with the time-course fluorescence profiles of the labelled V216C mutant. For penicillin G, the UV absorbance at 233 nm decreases as a function of time due to the enzymatic hydrolysis of the β-lactam ring (Supplementary Figure S8A available at http://www.bioscirep.org/bsr/034/bsr034e136add.htm). The hydrolytic reaction is virtually complete after 500 s. Interestingly, the fluorescence of the labelled V216C mutant is enhanced instantaneously upon addition of penicillin G and then declines gradually in a similar time interval (~500 s) (Figure S8B). For ampicillin, the fluorescence of the labelled V216C mutant and the UV absorbance of ampicillin both decrease in a similar time interval (~400 s) (Supplementary Figure S9A–9B available at http://www.bioscirep.org/bsr/034/bsr034e136add.htm). These observations indicate that the labelled V216C mutant gives stronger fluorescence upon binding to penicillin G and ampicillin to form ES* complexes and then restores its weak fluorescence as a result of the release of the hydrolysed products (ES*→E+P).

**Figure 6:**
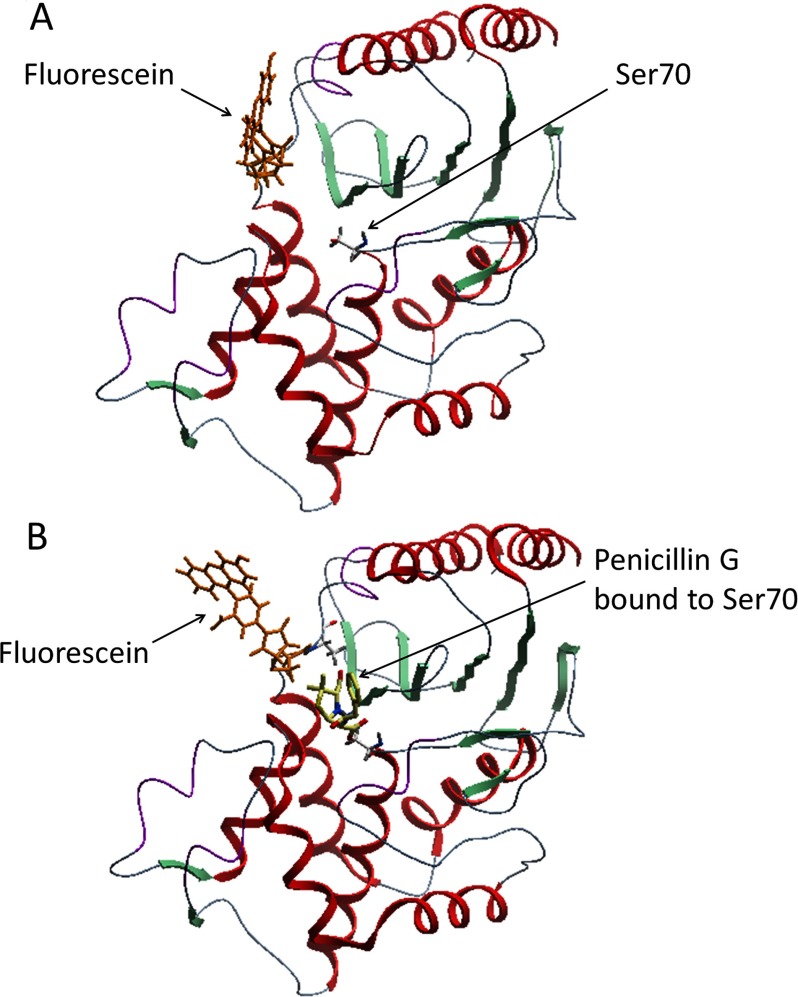
Molecular models of the fluorescein-labelled TEM-1 V216C mutant in the free-enzyme (E) and ES* states (**A**) Without penicillin G, the fluorescein molecule (orange) lies close to the active site of the enzyme. (**B**) With penicillin G, the fluorescein molecule (orange) stays away from the active site when penicillin G (yellow) binds to Ser^70^ in the active site. The ribbon diagrams represent the structure of the TEM-1 V216C mutant.

In order to examine the effect of drug binding at the active site on the conformation of the attached fluorescein molecule, we conducted molecular modelling on the free enzyme (E) and ES* states of the labelled V216C mutant with penicillin G as the substrate. Figure 6 shows the molecular models of the labelled V216C mutant with and without binding to penicillin G. In the free-enzyme state, the fluorescein molecule lies close to the active site (Figure 6A). Upon binding to penicillin G, the fluorescein molecule stays away from the active site (Figure 6B). This subtle conformational change is likely to keep the fluorescein molecule away from the residues around the active site, thus reducing their fluorescence quenching on the fluorescein molecule.

**Figure 7:**
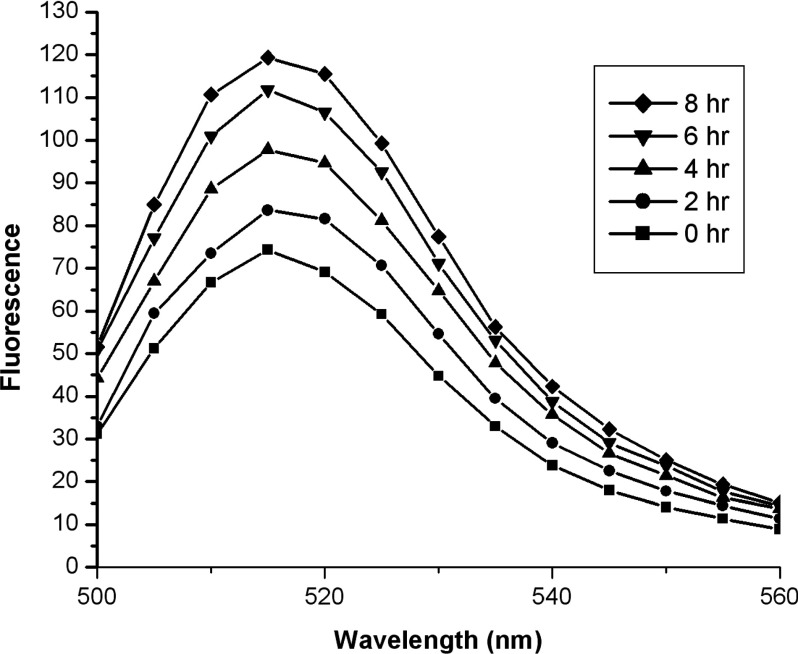
Time-course fluorescence measurements on the labelled V216C mutant in the presence of trypsin The fluorescence of the labelled V216C mutant increases in the course of trypsin digestion. The labelled V216C mutant (0.2 mg·ml^−1^) was mixed with trypsin (0.01 mg·ml^−1^) in 50 mM potassium phosphate buffer (pH 7.0).

We compared the fluorescence of the fluorescein molecule in the folded state of the labelled V216C mutant and its fragmented state after trypsin digestion. This comparative study can verify whether the fluorescein molecule fluoresces weakly when staying close to the active site of the enzyme (folded state) and gives stronger fluorescence when staying away from the active site (fragmented state). The digestion on the labelled V216C mutant by trypsin was first monitored by SDS/PAGE analysis. Supplementary Figure S10 (available at http://www.bioscirep.org/bsr/034/bsr034e136add.htm) shows the fluorescent image of the SDS/PAGE gel for the labelled V216C mutant before and after trypsin digestion. Before trypsin digestion, the labelled V216C mutant shows a green fluorescent band at the position corresponding to the undigested form (Figure S10). After incubation with trypsin for 8 h, some green fluorescent bands appear at lower positions, indicating that the labelled V216C mutant was cleaved into smaller peptide fragments by trypsin (Figure S10). We then examined the fluorescence of the labelled V216C mutant in response to trypsin digestion. Interestingly, the fluorescence of the labelled V216C mutant increases as a function of time during the course of trypsin digestion (Figure 7). This experimental observation implies that the fluorescein molecule is likely to experience fluorescence quenching when it stays close to the enzyme's active site in the folded state. Such fluorescence quenching is relieved when the fluorescein molecule stays away from the active site as a result of the trypsin digestion on the structure of the labelled V216C mutant, thus leading to the fluorescence enhancement of the fluorescein molecule. Taking the results of the ESI–MS, molecular modelling and trypsin digestion studies together, the fluorescein molecule is likely to lie close to the active site in the free enzyme state (E) and fluoresces weakly due to fluorescence quenching by the residues around the active site. Upon binding to β-lactam antibiotics, the fluorescein molecule is likely to stay away from the active site and experience weaker fluorescence quenching, thus giving stronger fluorescence in the substrate-bound state (ES and ES*). After enzymatic hydrolysis, the hydrolysed product (P) will be released from the active site, and therefore the fluorescein molecule will approach the active site again and restore its weak fluorescence.

### Conclusions

In summary, we have discovered that the Val^216^ residue in TEM-1 is a good site for the replacement with a cysteine and subsequent fluorophore labelling at Cys^216^ for the construction of a fluorescent drug sensor for *in vitro* drug screening. The labelled TEM-1 V216C mutant is catalytically active compared with the wild-type enzyme and can distinguish drug binders from non-binders and ‘drug aggregates’ by giving characteristic fluorescence profiles. The labelled TEM-1 V216C mutant can also fluorescently differentiate between potent and impotent β-lactam antibiotics, highlighting its potential use in the development of new-generation β-lactam antibiotics. With its catalytically active nature and effective fluorescent drug-sensing function, the labelled TEM-1 V216C mutant can mimic its wild-type form as a molecular drug target for *in vitro* drug screening. More importantly, given the high conservation of Val^216^ in the members of the TEM family, the combined strategy of Val^216^→Cys^216^ mutation and fluorophore labelling at Cys^216^ can be further extended to other clinically significant TEM variants (ESBL and IRT) for the construction of tailor-made *in vitro* drug screening tools. Such tools will greatly advance the drug discovery and development against clinically significant TEM-type β-lactamases. In this regard, the labelled TEM-type V216C mutants have the potential to act as high-throughput antibiotic screening tools against β-lactamase-producing bacteria with the aid of fluorescence microplate readers [41]. With β-lactamase-resistant antibiotics, the β-lactamase produced by bacteria will be inhibited, and therefore the labelled V216C mutant will receive intact antibiotics and give stronger fluorescence signals [41]. For β-lactamase-unstable antibiotics, they will be rapidly hydrolysed by the β-lactamase from bacteria, and therefore the labelled V216C mutant will be depleted of intact antibiotics and give much weaker fluorescence signals [41]. In addition to this drug screening function against antibiotic-resistant bacteria, the labelled TEM-type V216C mutants can mimic their wild-type forms to provide insights into the potency of new β-lactam antibiotics and their inactivation mechanisms by exhibiting characteristic fluorescence profiles [20]; the increasing and decreasing time-course fluorescence signals indicate the formation of ES* complex through acylation and the restoration of free enzyme (E) through deacylation, respectively [20]. The use of such fluorescence profiles will facilitate the design of new β-lactam antibiotics and β-lactamase inhibitors with stronger potency.

## Online data

Supplementary data
