# Supplementary material for: Fluorescent TEM-1 β-lactamase with wild-type activity as a rapid drug sensor for in vitro drug screening
Source: Biosci Rep. 2014 Sep 5;34(5):e00136. doi: 10.1042/BSR20140057 (PMC4155835; doi:10.1042/BSR20140057)
Supplement: Supplementary data [file bsr034e136add.pdf]

Biosci. Rep. (2014) / **34** / art:e00136 / doi 10.1042/BSR20140057

OPEN ACCESS

Original Paper

Bioscience Reports

www.bioscirep.org

Bioscience Reports

SUPPLEMENTARY DATA

Fluorescent TEM-1 β-lactamase with wild-type activity as a rapid drug sensor for *in vitro* drug screening

Wing-Lam Cheong*, Ming-San Tsang*, Pui-Kin So*, Wai-Hong Chung*, Yun-Chung Leung* and Pak-Ho Chan*†[1]

*State Key Laboratory of Chirosciences, Food Safety and Technology Research Centre, Department of Applied Biology and Chemical Technology, The Hong Kong Polytechnic University, Hung Hom, Kowloon, Hong Kong, People's Republic of China
†The Hong Kong Polytechnic University Shenzhen Research Institute, Shenzhen 518057, People's Republic of China

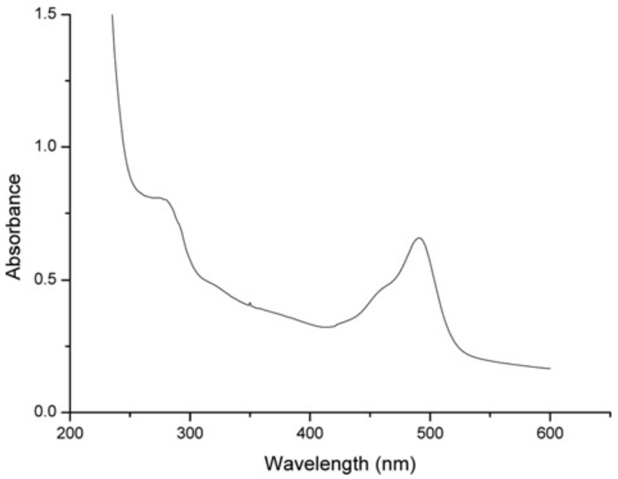

Figure S1 UV/Vis absorption spectrum of the fluorescein-labelled V216C mutant in 50 mM potassium phosphate buffer (pH 7.0)
[Labelled V216C] = 10 μM.

[1] To whom correspondence should be addressed (email: pak-ho.chan@polyu.edu.hk).

© 2014 The Author(s) This is an Open Access article distributed under the terms of the Creative Commons Attribution Licence (CC-BY) (http://creativecommons.org/licenses/by/3.0/) which permits unrestricted use, distribution and reproduction in any medium, provided the original work is properly cited.

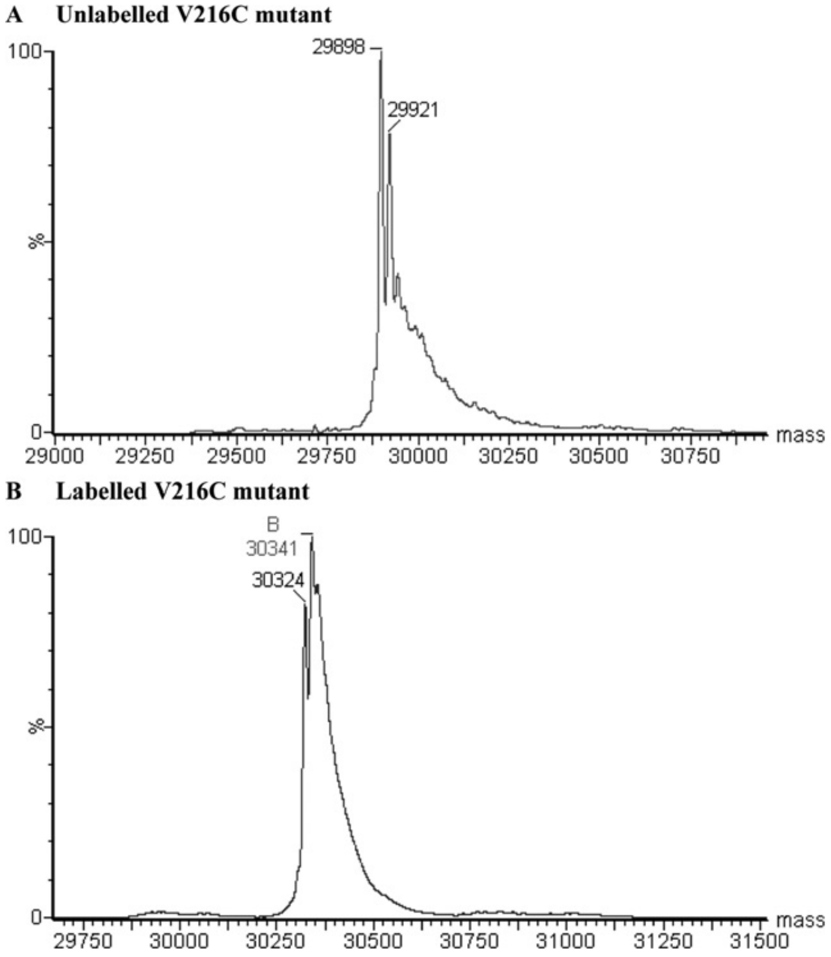

Figure S2 ESI mass spectra of the TEM-1 V216C mutant with and without labelling with fluorescein-5-maleimide
(**A**) Mass spectrum of the V216C mutant without fluorophore labelling. The mass peaks of 29898 and 29 921 kDa correspond to the V216C mutant and the [V216C + Na] adduct, respectively. (**B**) Mass spectrum of the V216C mutant with fluorophore labelling. The mass peaks of 30324 and 30341 Da correspond to the labelled V216C mutant and the [labelled V216C + H_2O] adduct, respectively.

© 2014 The Author(s) This is an Open Access article distributed under the terms of the Creative Commons Attribution Licence (CC-BY) (http://creativecommons.org/licenses/by/3.0/) which permits unrestricted use, distribution and reproduction in any medium, provided the original work is properly cited.

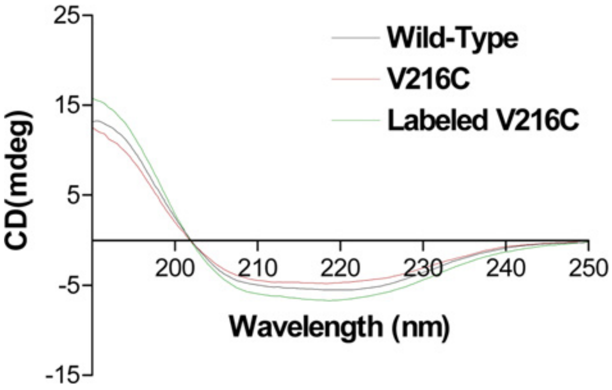

Figure S3 Far-UV CD measurements of the wild-type and mutant forms of the TEM-1 β-lactamase
Far-UV CD signals: wild-type TEM-1 (black line), unlabelled V216C (red line) and labelled V216C (green line).

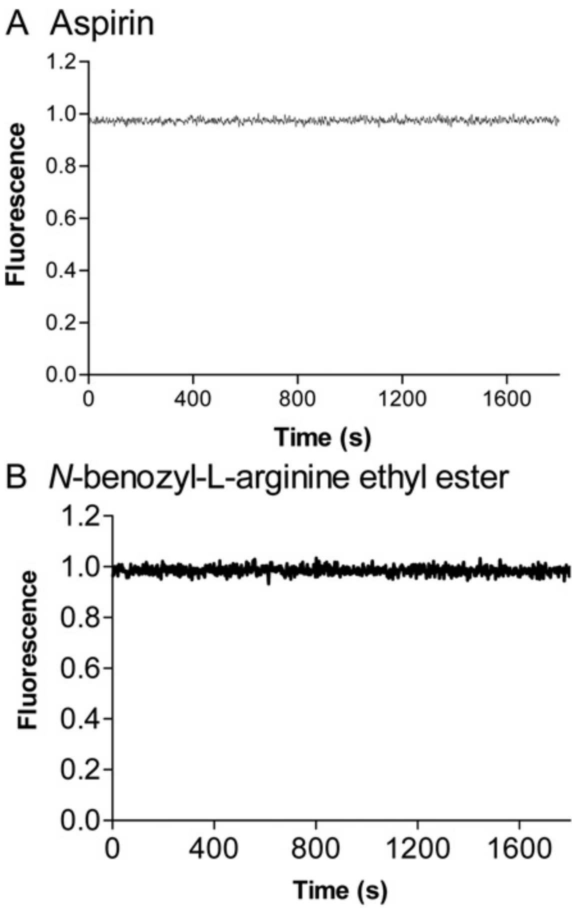

Figure S4 Time-course fluorescence measurements of the labelled V216C mutant with non-binders
(**A**) fluorescence signals of the labelled V216C mutant (20 nM) with 1 mM aspirin; (**B**) fluorescence signals of the labelled V216C mutant (20 nM) with 1 mM BAEE. Excitation: 494 nm. Buffer: 50 mM potassium phosphate (pH 7.0).

© 2014 The Author(s) This is an Open Access article distributed under the terms of the Creative Commons Attribution Licence (CC-BY) (http://creativecommons.org/licenses/by/3.0/) which permits unrestricted use, distribution and reproduction in any medium, provided the original work is properly cited.

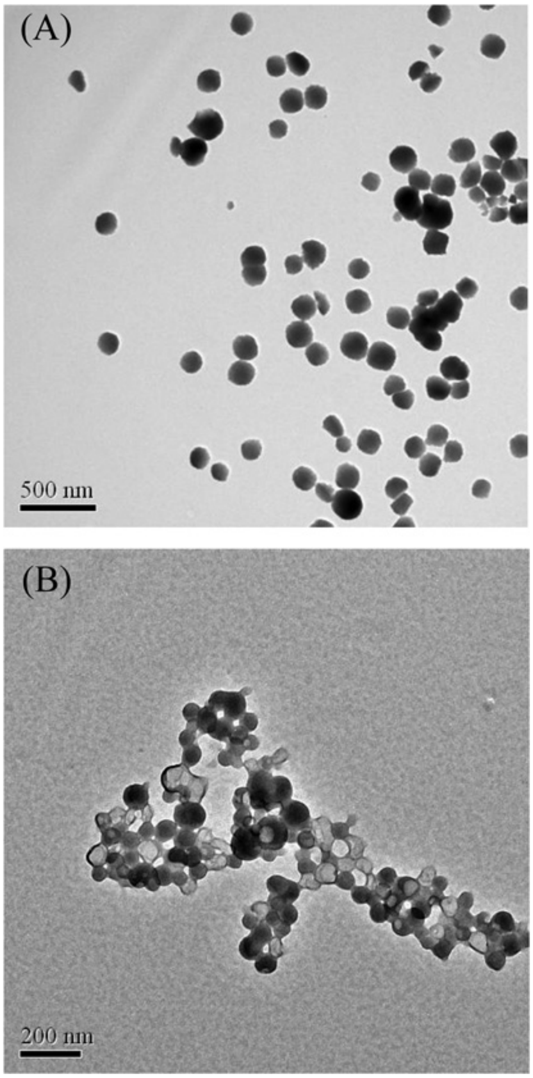

Figure S5 Transmission electron microscopic images of (A) Congo red (10 μM) and (B) TIPP (10 μM) in the presence of the labelled V216C mutant (20 nM)

Aggregates were formed by Congo red and TIPP under aqueous conditions.

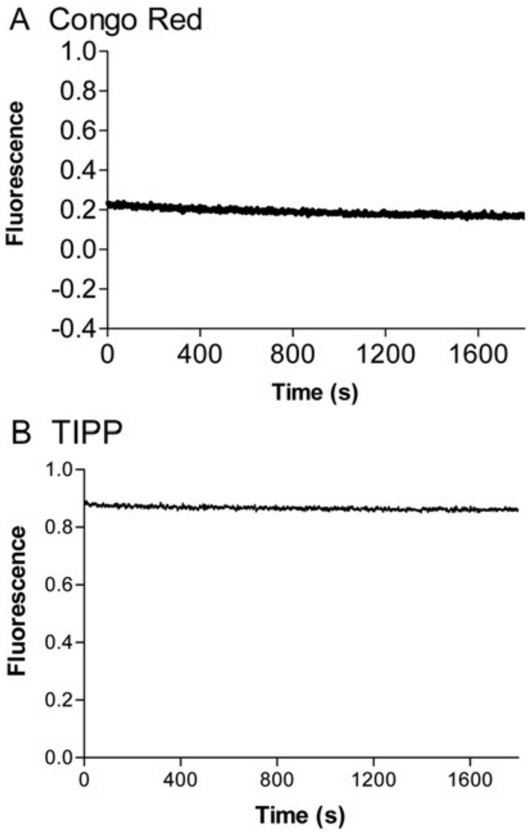

Figure S6 Time-course fluorescence measurements of the labelled V216C mutant in the presence of 'drug aggregates'

(**A**) fluorescence signals of the labelled V216C mutant (20 nM) in the presence of 10 μM Congo red; (**B**) fluorescence signals of the labelled V216C mutant (20 nM) in the presence of 10 μM TIPP. Buffer: 50 mM potassium phosphate (pH 7.0). Congo red and TIPP formed aggregates under such aqueous conditions (Figure S5).

© 2014 The Author(s) This is an Open Access article distributed under the terms of the Creative Commons Attribution Licence (CC-BY) (http://creativecommons.org/licenses/by/3.0/) which permits unrestricted use, distribution and reproduction in any medium, provided the original work is properly cited.

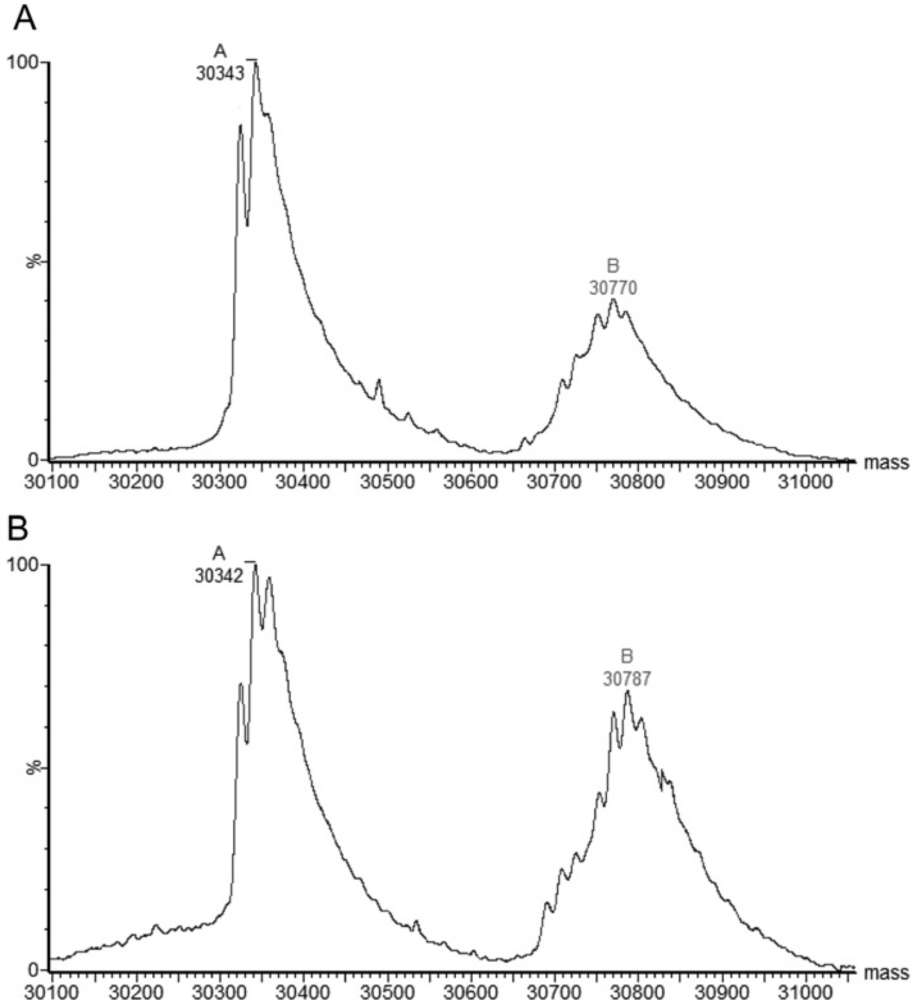

Figure S7 ESI mass spectra of the labelled V216C mutant (5 μM) with cefoxitin (10 mM) at various time intervals
(**A**) $t = 1$ min and (**B**) $t = 10$ min. Buffer: 20 mM ammonium acetate solution (pH 7.0). Peaks A and B correspond to the labelled V216C mutant and the covalent labelled V216C-cefoxitin complex, respectively.

© 2014 The Author(s) This is an Open Access article distributed under the terms of the Creative Commons Attribution Licence (CC-BY) (http://creativecommons.org/licenses/by/3.0/) which permits unrestricted use, distribution and reproduction in any medium, provided the original work is properly cited.

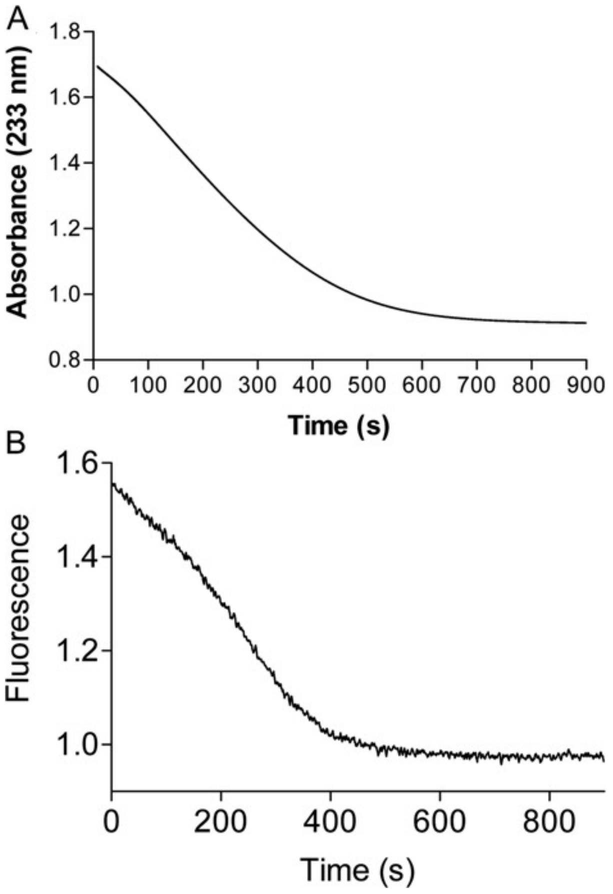

Figure S8 Time-course fluorescence and UV absorbance measurements of the labelled V216C mutant with penicillin G
(**A**) UV absorbance of penicillin G at different time intervals; (**B**) fluorescence signals of the labelled V216C mutant at different time intervals. [Labelled V216C] = 20 nM; [Penicillin G] = 1 mM. Buffer: 50 mM potassium phosphate buffer (pH 7.0).

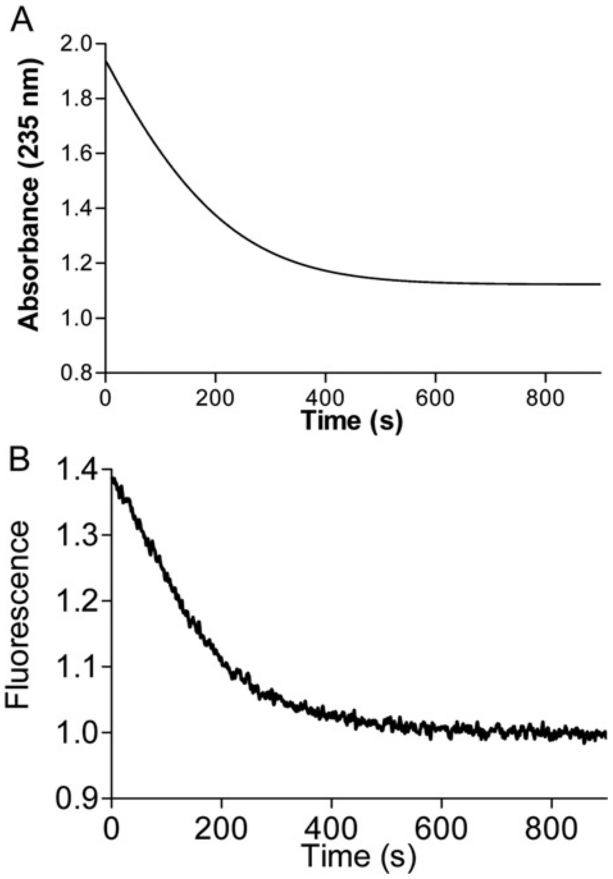

Figure S9 Time-course fluorescence and UV absorbance measurements of the labelled V216C mutant with ampicillin
(**A**) UV absorbance of ampicillin at different time intervals; (**B**) fluorescence signals of the labelled V216C mutant at different time intervals. [Labelled V216C] = 20 nM; [Ampicillin] = 1 mM. Buffer: 50 mM potassium phosphate buffer (pH 7.0).

© 2014 The Author(s) This is an Open Access article distributed under the terms of the Creative Commons Attribution Licence (CC-BY) (http://creativecommons.org/licenses/by/3.0/) which permits unrestricted use, distribution and reproduction in any medium, provided the original work is properly cited.

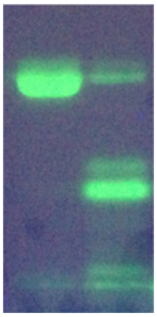

Figure S10 SDS/PAGE analysis of the labelled V216C mutant with and without trypsin digestion
The SDS/PAGE gel was illuminated with the UV light. (Left) the labelled V216C mutant without trypsin digestion; (Right) the labelled V216C mutant after trypsin digestion.

Received 4 April 2014/7 July 2014; accepted 28 July 2014

Published as Immediate Publication 30 July 2014, doi 10.1042/BSR20140057

© 2014 The Author(s) This is an Open Access article distributed under the terms of the Creative Commons Attribution Licence (CC-BY) (http://creativecommons.org/licenses/by/3.0/) which permits unrestricted use, distribution and reproduction in any medium, provided the original work is properly cited.
